# CDK8/19 inhibition induces premature G1/S transition and ATR-dependent cell death in prostate cancer cells

**DOI:** 10.18632/oncotarget.24414

**Published:** 2018-02-06

**Authors:** Akito Nakamura, Daisuke Nakata, Yuichi Kakoi, Mihoko Kunitomo, Saomi Murai, Shunsuke Ebara, Akito Hata, Takahito Hara

**Affiliations:** ^1^ Oncology Drug Discovery Unit, Takeda Pharmaceutical Company Limited, Kanagawa, Japan; ^2^ Bio Molecular Research Laboratories, Takeda Pharmaceutical Company Limited, Kanagawa, Japan

**Keywords:** CDK19, CDK8, prostate cancer, cell cycle, ATR

## Abstract

The CDK8/19 kinase module comprises a subcomplex that interacts with the Mediator complex and regulates gene expression through phosphorylation of transcription factors and Mediator subunits. Mediator complex subunits have been increasingly implicated in cancer and other diseases. Although high expression of CDK8/19 has been demonstrated in prostate cancer, its function has not been thoroughly examined. Here we report that CDK8/19 modulates the gene expression of cell cycle regulators and thereby maintains the proper G1/S transition in prostate cancer cells. We show that highly selective CDK8/19 inhibitors exerted anti-proliferative activity in prostate cancer cells both *in vitro* and *in vivo*. In CDK8/19 inhibitor-sensitive prostate cancer cells, the compounds reduced the population of G1 phase cells and elevated that of S phase cells through the modulation of G1/S transition regulators at the level of mRNA expression. Furthermore, the premature G1/S transition induced a DNA damage response that was followed by ATR-dependent and caspase-independent cell death. These findings suggest a novel role of CDK8/19 in transcription-mediated cell cycle control, albeit with possible contribution of other proteins inhibited by the compounds. Our data provide a rationale for further investigation of CDK8/19 inhibitors as a new therapeutic approach to prostate cancer.

## INTRODUCTION

The cell cycle is controlled by CDKs and their binding partners known as cyclins. Proper progression of the cell cycle is monitored by checkpoints that recognize defects during multiple stages in the cell cycle such as in DNA synthesis and chromosome segregation. Activation of these checkpoints induces cell cycle arrest through the modulation of CDK activity [1]. Although CDKs were originally characterized by their roles in cell cycle regulation, several members of this family have direct functions in the regulation of RNA polymerase II (Pol II) activity, of which the best known representatives are CDK7, CDK8, and CDK9 [2]. Among these transcriptional CDKs, CDK8 and its closely related paralog CDK19 constitute alternative subunits of the regulatory CDK module of the Mediator complex, which links transcription factors with Pol II [3]. The Mediator complex also has a role in transcription elongation and pausing as well as in chromatin remodeling, facilitating the formation of enhancer-promoter gene loops [4, 5]. This complex can influence cell identity by regulating genes associated with super-enhancers, which are composed of a large cluster of enhancers [4, 5]. By functioning as a hub for input from numerous signaling pathways, the Mediator complex including CDK8/19 activates or represses transcription cycles [3, 4]. Notably, the role of the CDK module in transcription is thought to be context-dependent, such that its biological function may vary among different cell types or in response to distinct stimuli.

A certain subset of CDKs (CDK1, CDK2, CDK4, and CDK6) and the corresponding cyclins is directly involved in the control of the cell cycle. In contrast to these members of the CDK family, the cyclin subunits of transcriptional CDKs do not show oscillating patterns in their protein levels during the cell cycle. However, several studies have recently demonstrated the direct and indirect role of transcriptional CDKs in cell cycle control [6, 7]. Different stages (e.g., G1/S and G2/M transition) in the cell cycle and the related pathways (e.g., p53/p21, Wnt/β-catenin, and Rb/E2F) have been suggested to require CDK8 for proper function [8–13]. Thus, these recent findings begin to clarify a plausible role for the CDK module in Mediator as a hub for integrating transcription regulation with cell cycle control.

Notably, several subunits of the Mediator complex have been implicated in the pathogenesis of human diseases including cancer [4, 14, 15], for which most evidence has been obtained regarding the involvement of CDK8. In particular, amplification and overexpression of CDK8 have been found in colon, breast, and prostate cancers [14, 16–18], which underlies the marked attention that CDK8 has attracted as a potential target for cancer therapy by small molecule inhibitors [19–21]. In comparison, although information on CDK19 has been limited, the *CDK19* gene has been recently implicated in prostate cancer [14, 22]. However, despite their importance as potential therapeutic targets for prostate cancer, the function and importance of CDK8/19 in prostate cancer remain poorly understood.

To address this deficit, in this preclinical study, we used both small molecule inhibitors of CDK8/19 and genetic approaches to investigate the dependence of prostate cancer cells on CDK8/19 activity. Furthermore, we explored the biological roles of CDK8/19 in prostate cancer cells as well.

## RESULTS

### Anti-proliferative activity of CDK8/19 inhibitors in prostate cancer cells

**Figure 1:**
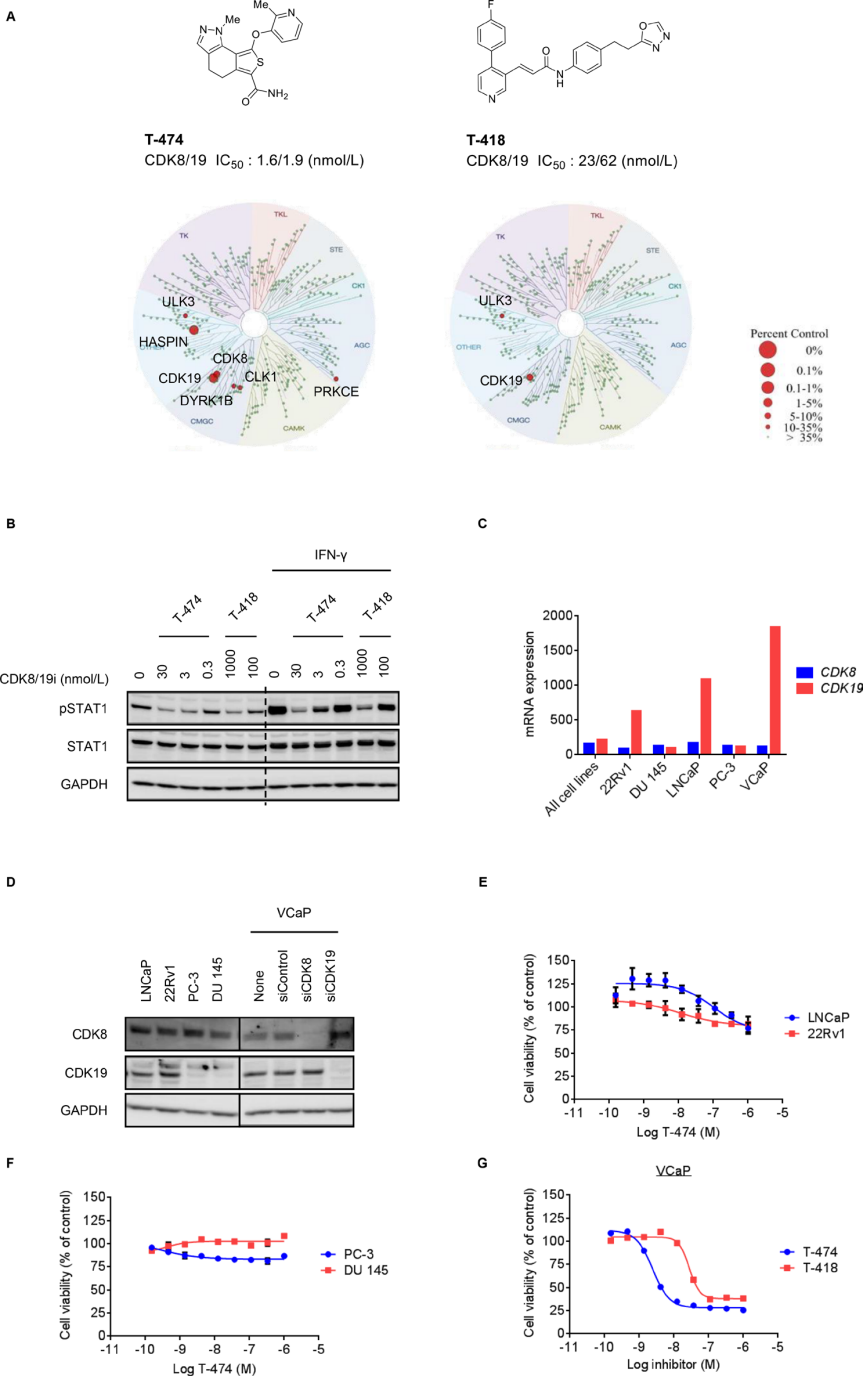
Anti-proliferative activity of CDK8/19 inhibitors in prostate cancer cells (**A**) Compound structure, potency, and kinase selectivity of T-474 or T-418. Kinase selectivity profiling was performed using 300 nmol/L T-474 or T-418. (**B**) VCaP cells were treated with T-474 or T-418 together with 10 ng/mL IFN-γ as indicated for 30 minutes. Cell lysates were analyzed by western blot. (**C**) mRNA expression of CDK8 or CDK19 in prostate cancer cell lines (CCLE). (**D**) Western blot of CDK8 or CDK19 in prostate cancer cell lines. VCaP cells were transfected with siRNA as indicated for 72 hours. Cell lysates were analyzed by western blot. The relative band intensities of CDK8 or CDK19 were quantified and are indicated as percentage (%) of control (non-treated VCaP cells). An arrow indicates the expected position of bands derived from CDK19. (**E**) LNCaP or 22Rv1 cells were treated with T-474 as indicated for 9 days (*N* = 3, mean with *SD*). (**F**) PC-3 or DU 145 cells were treated with T-474 as indicated for 6 days (*N* = 3, mean with *SD*). (**G**) VCaP cells were treated with T-474 or T-418 as indicated for 7 days (*N* = 2, mean). Cell viability was measured.

To accurately explore the function of CDK8 and CDK19, we used two structurally differentiated compounds, both of which potently inhibit CDK8 and CDK19, in enzyme assays (T-474; CDK8/19 IC_50_ = 1.6/1.9 nmol/L, T-418; CDK8/19 IC_50_ = 23/62 nmol/L) (Figure 1A). In a panel of 456 kinases, both compounds showed marked kinase selectivity (Figure 1A and Supplementary Tables 1 and 2). Kinases inhibited by >80% in response to 300 nM T-474 were limited to CDK19 (99% inhibition), Haspin (99% inhibition), and CDK8 (90% inhibition). CDK19 was the only kinase that was inhibited by >80% in response to 300 nM T-418 (94% inhibition) (Supplementary Tables 1 and 2). In VCaP prostate cancer cells, treatment with T-474 or T-418 suppressed the phosphorylation of the known CDK8 substrate STAT1 at Ser727 both in the absence and in the presence of IFN-γ (Figure 1B), which stimulates CDK8-mediated STAT1 phosphorylation [23]. Furthermore, T-474 treatment reduced Wnt/β-catenin-dependent transcriptional activity in SW480 colon cancer cells as reported previously (Supplementary Figure 1) [17].

We then investigated the expression of CDK8 and CDK19 in several commercially available prostate cancer cell lines. In accordance with previous reports [14], CDK19 was highly expressed in some prostate cancer cells at the mRNA and protein levels (Figure 1C, 1D, and Supplementary Figure 2). We observed that CDK8 protein levels were moderately elevated in CDK19-depleted cells (Figure 1D and Supplementary Figure 2). Notably, similar compensatory effects in paralogs have been reported previously [24]. CDK8/19 inhibition did not obviously impact proliferation of LNCaP, 22Rv1, PC-3, or DU 145 cells (Figure 1E and 1F), whereas we observed that treatment with T-474 or T-418 substantially inhibited the proliferation of VCaP cells (Figure 1G). Furthermore, in VCaP cells, knockdown of CDK8 or CDK19 by siRNA did not obviously impact the cell proliferation (Supplementary Figure 3A). Specifically, only one of four CDK19 siRNAs substantially suppressed cell proliferation; however, the effects appeared to be off-target considering the limited knockdown efficiency (Supplementary Figure 3A). Importantly, the simultaneous knockdown of CDK8 and CDK19 suppressed the proliferation of VCaP cells (Supplementary Figure 3B). These results suggest that inhibition of both CDK8 and CDK19 is essential for suppression of VCaP cell proliferation.

### Effects of CDK8/19 inhibition on cell cycle progression

**Figure 2:**
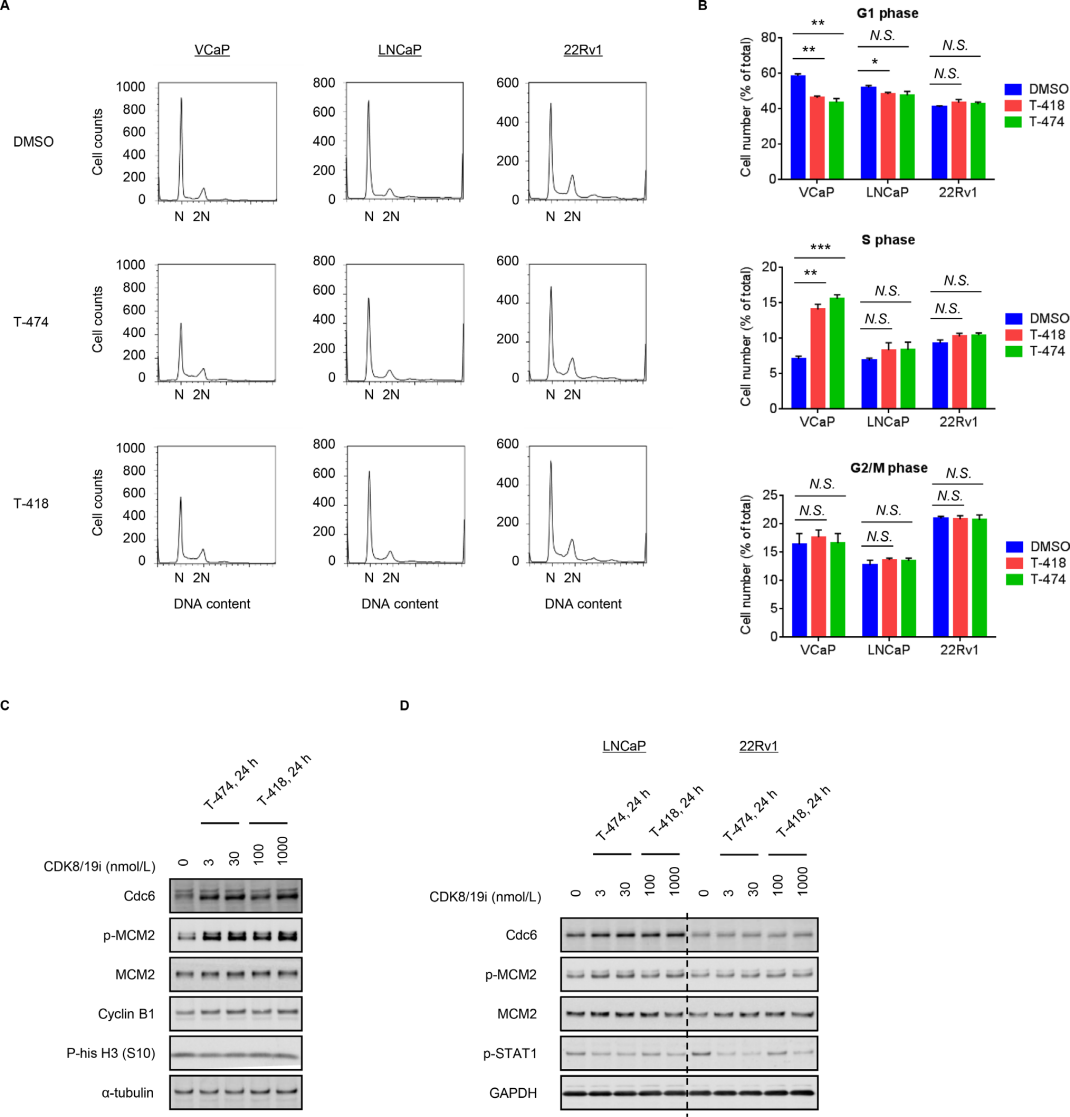
Effects of CDK8/19 inhibitors on cell cycle progression (**A–B**) VCaP, LNCaP, and 22Rv1 cells were treated with 30 nmol/L T-474 or 1000 nmol/L T-418 for 24 hours. Cell cycle profile was analyzed using a flow cytometer. (A) Histogram. (B) Bars show the population of cells in G1, S, and G2/M phase (*N* = 3, mean with *SD*). ^*^P < 0.01; ^**^P < 0.001; and ^***^P < 0.0001. (**C**) VCaP cells were treated with T-474 or T-418 as indicated for 24 hours. (**D**) LNCaP and 22Rv1 cells were treated with T-474 or T-418 as indicated for 24 hours. Cell lysates were analyzed by western blot. *N.S.*, not significant (*P* > 0.05).

Given that CDK8/19 forms a subcomplex of Mediator, it was plausible that inhibition of CDK8/19 might affect the gene expression pattern. To understand the mechanism of action, we performed a microarray analysis in CDK8/19 inhibitor-sensitive VCaP cells. A comprehensive evaluation of transcriptional changes using a parametric analysis of gene set enrichment (PAGE) revealed that down-regulated genes following T-474 treatment were enriched for multiple processes related to cell migration (Table 1), in accordance with a previous report [14]. Notably, the down-regulated processes included regulation of androgen receptor (AR) activity, which is important for pathophysiology of prostate cancer. However, we also observed that treatment with T-474 or T-418 reduced the mRNA expression of *KLK3* (a well-known AR target gene) in LNCaP and 22Rv1 cells (Supplementary Figure 4). Considering that treatment with CDK8/19 inhibitors suppressed the proliferation of VCaP but not LNCaP and 22Rv1 cells, the effects on AR signaling are considered unlikely to be involved in the anti-proliferative activity. In comparison, the up-regulated processes following CDK8/19 inhibitor treatment were primarily related to cell cycle progression and DNA replication (Table 1). Flow cytometric analysis revealed that the population of G1 phase cells was decreased and that of S phase cells was increased in VCaP cells treated with T-474 or T-418 (Figure 2A and 2B). In contrast, in CDK8/19 inhibitor-insensitive LNCaP and 22Rv1 cells, changes in the cell cycle profile following CDK8/19 inhibitor treatment were less obvious (Figure 2A and 2B). Consistent with these findings, treatment with the CDK8/19 inhibitors induced Cdc6 expression and MCM2 phosphorylation in VCaP cells (Figure 2C), both of which are implicated in DNA replication [25]. Conversely, the expression of Cdc6 and the phosphorylation of MCM2 were not affected by treatment with either compound in LNCaP and 22Rv1 cells (Figure 2D), although a reduction of STAT1 phosphorylation was detected (Figure 2D) as also observed in VCaP cells (Figure 1B). These results indicate that CDK8/19 inhibition decreases the population of G1 cells and increases that of S phase cells in CDK8/19 inhibitor-sensitive VCaP cells.

**Table 1:** Pathway analysis of down/up-regulated genes in VCaP cells following CDK8/19 inhibition for 24 hours with 30 nmol/L T-474

TOP 10 down-regulated pathways [gene number]	*Z*-score	*P*-value	Number of hit genes
Unfolded Protein Response [24]	6.70	0.0000	19
Activation of Chaperones by IRE1alpha [13]	6.61	0.0000	10
Regulation of Androgen receptor activity [63]	5.56	0.0000	45
Regulation of actin cytoskeleton [396]	5.05	0.0000	152
Sema4D in semaphorin signaling [67]	4.19	0.0000	20
Leukocyte transendothelial migration [208]	4.10	0.0000	77
Sema4D induced cell migration and growth-cone collapse [56]	4.08	0.0000	16
a6b1 and a6b4 Integrin signaling [40]	3.90	0.0001	13
Lysosome [186]	3.82	0.0001	106
Vasopressin-regulated water reabsorption [79]	3.76	0.0001	40

TOP 10 up-regulated pathways [gene number]	*Z*-score	*P*-value	Number of hit genes
Cell Cycle, Mitotic [401]	–14.87	0.0000	294
G1/S Transition [115]	–12.92	0.0000	96
E2F transcriptional targets at G1/S [24]	–12.88	0.0000	17
E2F mediated regulation of DNA replication [32]	–12.60	0.0000	23
G2/M Checkpoints [47]	–12.30	0.0000	38
DNA strand elongation [39]	–12.12	0.0000	31
Gene Expression [549]	–11.94	0.0000	407
DNA replication [42]	–11.60	0.0000	36
Pyrimidine biosynthesis (interconversion) [14]	–11.54	0.0000	10
Glucose Regulation of Insulin Secretion [215]	–11.48	0.0000	143

### Induction of DNA damage followed by ATR-dependent cell death by CDK8/19 inhibition

**Figure 3:**
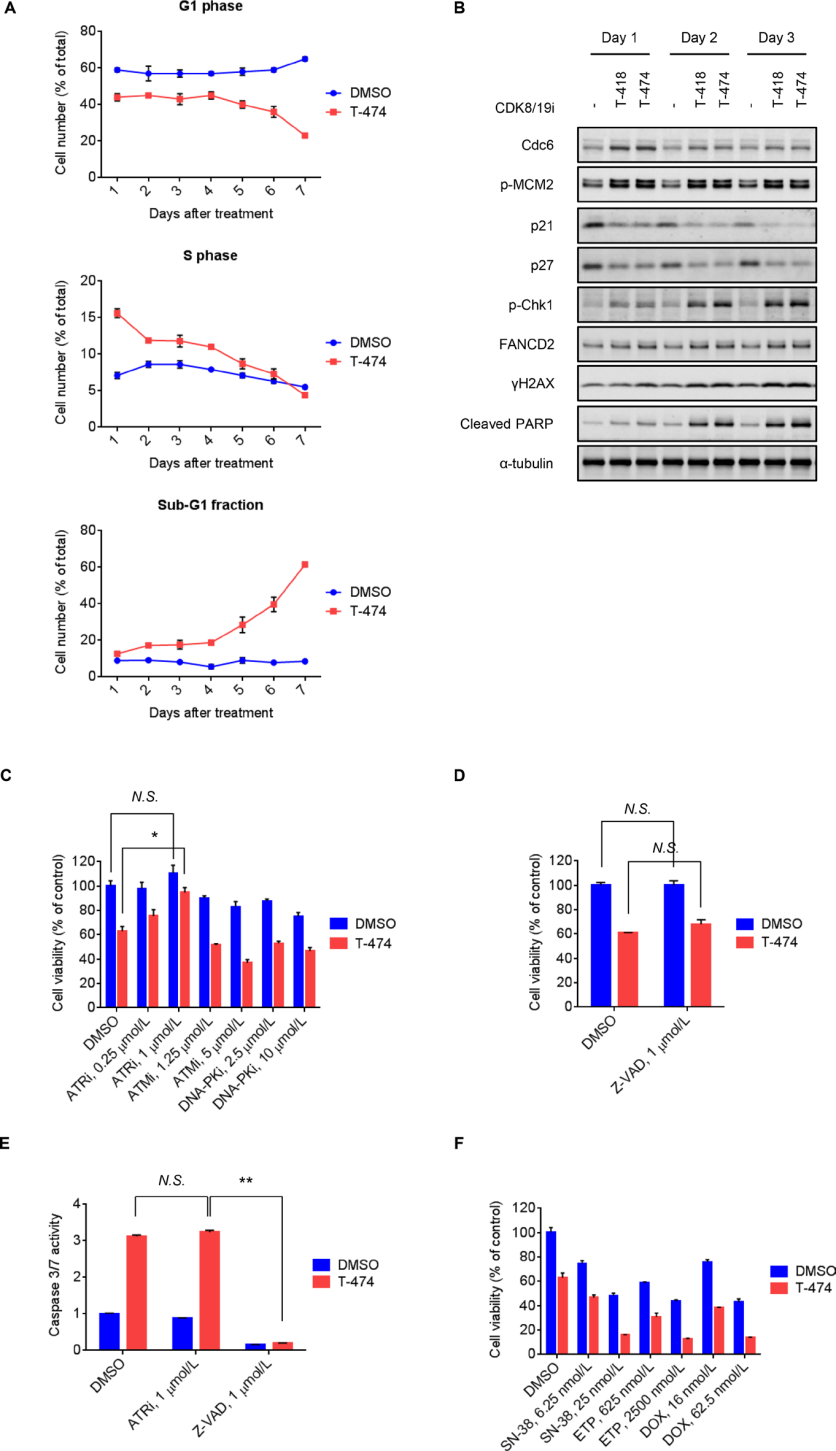
Induction of DNA damage response followed by ATR-dependent cell death mediated by CDK8/19 inhibition (**A**) VCaP cells were treated with 30 nmol/L T-474 for 1–7 days. Cell cycle profile was analyzed using a flow cytometer. Line graphs show the population of cells in G1, S, and sub-G1 phase (*N* = 3, mean with *SD*). (**B**) VCaP cells were treated with 30 nmol/L T-474 or 300 nmol/L T-418 for 1–3 days. Cell lysates were analyzed by western blot. (**C**) VCaP cells were treated with T-474 together with the ATR inhibitor VE-821, ATM inhibitor KU-55933, or DNA-PK inhibitor NU7026 as indicated for 4 days. (**D**) VCaP cells were treated with T-474 together with Z-VAD as indicated for 4 days. Cell viability was measured (*N* = 3, mean with *SD*). (**E**) VCaP cells were treated with T-474 together with ATRi (VE-821) or Z-VAD as indicated for 4 days. Caspase 3/7 activity was measured (*N* = 3, mean with *SD*). ^*^P < 0.01 and ^**^P < 0.000001. (**F**) VCaP cells were treated with T-474 together with SN-38, ETP (etoposide), or DOX (doxorubicin) as indicated for 4 days. Cell viability was measured (*N* = 3, mean with *SD*). *N.S.*, not significant (*P* > 0.05).

Next, to investigate the cell fate of CDK8/19 inhibitor-treated VCaP cells, we examined the long-term effects of CDK8/19 inhibition. Flow cytometric analysis revealed a sustained decrease in G1 phase and an increase in S phase populations following T-474 treatment (Figure 3A). Consistent with this phenomenon, treatment with T-474 or T-418 not only increased Cdc6 expression and MCM2 phosphorylation, but also decreased the expression of p21 and p27 (Figure 3B), which are inhibitors of the CDK2/4/6-mediated G1/S transition [26]. Notably, western blot analysis revealed that treatment with the CDK8/19 inhibitors induced markers of the DNA damage response (DDR) [27] such as Chk1 phosphorylation, mono-ubiquitination of FANCD2 (as indicated by a reduced mobility of bands), and γH2AX, at later time points relative to the changes in cell cycle regulators (Figure 3B). We therefore next examined whether the activation of DDR is involved in the anti-proliferative activity of CDK8/19 inhibitors. As DDR is known to be regulated by sensor kinases such as ATR, ATM, and DNA-PK [27], we utilized inhibitors of these kinases [28–30]. Treatment with the ATR inhibitor VE-821 significantly reversed the reduced cell viability mediated by CDK8/19 inhibition in VCaP cells, whereas treatment with the ATM inhibitor KU-55933 and DNA-PK inhibitor NU7026 did not (Figure 3C).

In flow cytometric analysis, we noted that T-474 treatment elevated the sub-G1 fraction (Figure 3A), which is indicative of apoptotic cell death. Treatment with CDK8/19 inhibitors also induced cleavage of PARP (Figure 3B); however, caspase inhibitor Z-VAD treatment did not reverse the reduced cell viability caused by CDK8/19 inhibition (Figure 3D), suggesting that CDK8/19 inhibition causes caspase activation but leads to caspase-independent cell death. In addition, despite its rescue effects on cell viability, the ATR inhibitor treatment did not prevent the caspase activation mediated by CDK8/19 inhibition (Figure 3E), further supporting the notion that the caspase activation by CDK8/19 inhibitors does not contribute to their cell-killing effects.

Previous findings on topoisomerase inhibitor-mediated replication stress and DNA damage [31, 32] prompted us to test the combination treatment of CDK8/19 inhibitors with topoisomerase inhibitors. CDK8/19 inhibitor-treated VCaP cells were more sensitive to the topoisomerase inhibitors SN-38 (an active metabolite of irinotecan), etoposide, and doxorubicin (Figure 3F and Supplementary Figure 5), probably owing to the accumulation of DNA damage caused by the effects of the drug combination.

### Premature G1/S transition by CDK8/19 inhibition

The reduction of G1 phase cells and accumulation of S phase cells can be caused by the delay of S phase progression or the acceleration of G1/S transition. To address whether CDK8/19 inhibition directly modulates S phase progression, we performed EdU pulse chase analysis in which cells were pre-treated with CDK8/19 inhibitors and then pulse-labeled with EdU in the presence of CDK8/19 inhibitors. T-474 treatment did not affect the population of early S phase cells in VCaP cells 0–10 hours after the release of EdU (Supplementary Figure 6), indicating that CDK8/19 inhibition does not directly impact S phase progression.

**Figure 4:**
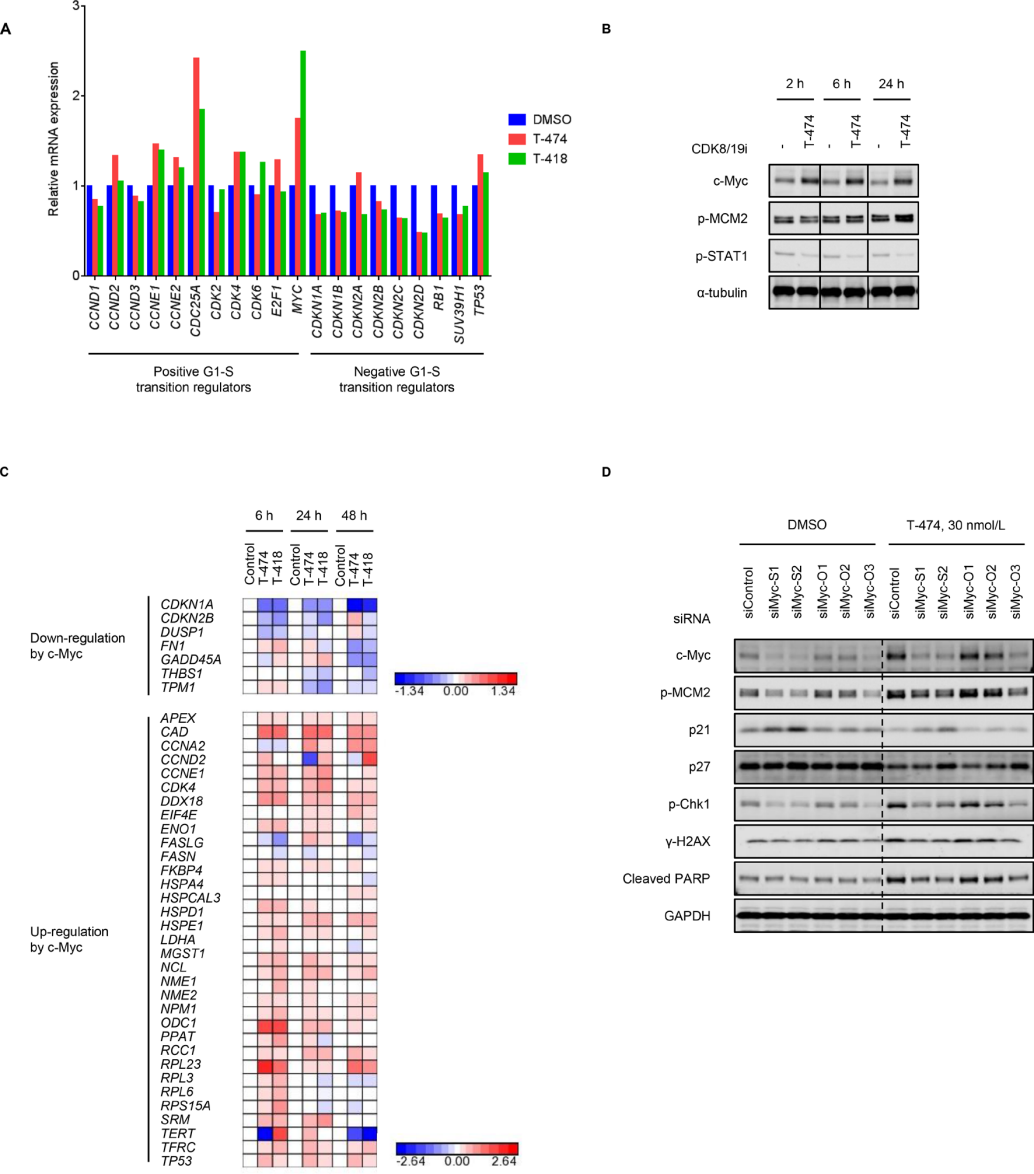
Aberrant G1/S transition by CDK8/19 inhibition (**A**) VCaP cells were treated with 30 nmol/L T-474 or 1000 nmol/L T-418 for 6 hours. Gene expression level was measured by microarray. Color bars show the relative gene expression of previously validated G1/S transition regulators (*N* = 2, mean). (**B**) VCaP cells were treated with 30 nmol/L T-474 as indicated for 2, 6, or 24 hours. Cell lysates were analyzed by western blot. Individual blots with dividing lines are combined from a single electrophoresis gel. (**C**) VCaP cells were treated with 30 nmol/L T-474 or 1000 nmol/L T-418 for 6, 24, or 48 hours. Gene expression level was measured by microarray. Color bars show the relative expression of previously validated c-Myc downstream genes (*N* = 2). (**D**) VCaP cells were transfected with siRNA as indicated for 48 hours and then treated with 30 nmol/L T-474 for 48 hours. Cell lysates were analyzed by western blot.

The transition from G1 phase to S phase is tightly regulated by various regulators [1]. As CDK8/19 can impact gene expression patterns as Mediator-associated kinases, we examined whether treatment with CDK8/19 inhibitors might affect the gene expression of the G1/S transition regulators. To analyze the gene expression changes that occurred prior to the changes in cell cycle profile, we performed microarray analysis using VCaP cells treated with CDK8/19 inhibitors for 6 hours. Treatment with CDK8/19 inhibitors not only increased the mRNA expression of positive regulators (e.g., CDC25A, cyclin E1, or c-Myc) but also decreased that of negative regulators (e.g., p19, p21, or p27) of the G1/S transition (Figure 4A). Western blot analysis revealed that T-474 treatment elevated c-Myc protein expression at least from 2 hours after compound treatment, whereas an increase in MCM2 phosphorylation was induced by the 24-hour treatment but not by the 2- or 6-hour treatment (Figure 4B). The results indicate that the gene expression changes in G1/S transition regulators would be earlier events. Notably, c-Myc upregulation following CDK8/19 inhibitor treatment was not observed in CDK8/19 inhibitor-insensitive LNCaP and 22Rv1 cells (Supplementary Figure 7). As the post-translational regulation of c-Myc expression has been well investigated [33], we also examined the turnover of c-Myc protein. The normalized c-Myc protein level in T-474-treated cells was maintained somewhat longer than in control cells, in the presence of the protein translation inhibitor cycloheximide (Supplementary Figure 8), suggesting that CDK8/19 inhibition delays the degradation of c-Myc protein, albeit with moderate effects. Given that T-474 treatment clearly increased *MYC* mRNA levels (Figure 4A), CDK8/19 may regulate c-Myc expression mainly transcriptionally but also post-transcriptionally to a certain degree. Consistent with the upregulation of c-Myc expression, we observed CDK8/19 inhibitor-mediated gene expression changes in previously identified c-Myc downstream effectors [34] (Figure 4C).

A shortened G1 phase and the fast transition through G1 phase enables cells to enter the S phase in the presence of unrepaired DNA damage followed by ATR activation [35]. We therefore investigated whether aberrant G1/S transition contributes to the induction of DDR in CDK8/19 inhibitor-treated cells by performing siRNA experiments. Western blot analysis revealed that c-Myc depletion reversed the changes in cell cycle regulator expression and the activation of DDR following T-474 treatment (Figure 4D). We confirmed that c-Myc knockdown did not obviously impact cell viability at that time point (Supplementary Figure 9), precluding the possibility that the effects of c-Myc knockdown on cell proliferation alleviated the effects of CDK8/19 inhibitors on the cell cycle. Furthermore, ATR inhibitor treatment did not affect the population of G1 or S phase cells when combined with CDK8/19 inhibition (Supplementary Figure 10), indicating that the DDR activation is not responsible for the aberrant cell cycle profile. These results suggest that CDK8/19 inhibitor-mediated gene expression changes accelerate the G1/S transition and thereby induce DDR.

### *In vivo* antitumor activity of CDK8/19 inhibition

**Figure 5:**
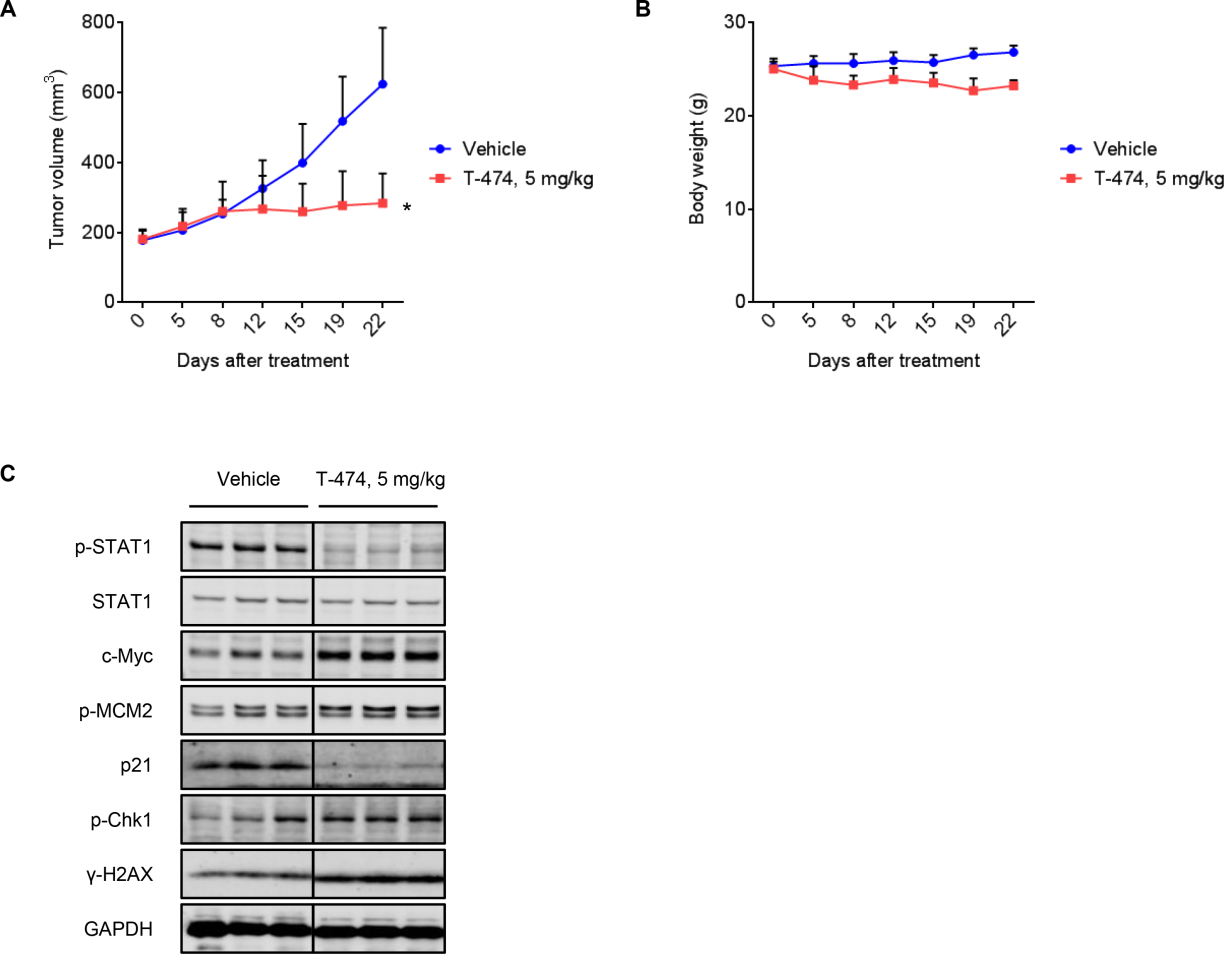
*In vivo* antitumor activity of CDK8/19 inhibition (**A–B**) Mice bearing VCaP xenografts were treated once daily with T-474 as indicated for 3 weeks. (A) Tumor growth curves. (B) Body weight change. Data represent mean tumor volume or body weight (*N* = 6, mean with *SD*). Day 0 indicates the beginning of treatment. ^*^P < 0.01 (Welch’s *t*-test, compared with vehicle-treated control). (**C**) Mice bearing VCaP xenografts were treated once daily with T-474 as indicated for 3 days. Tumor xenografts were harvested 8 hours after the final dosing and analyzed by western blot (*N* = 3). Individual blots with dividing lines are combined from a single electrophoresis gel.

Next, we examined the *in vivo* activity of a CDK8/19 inhibitor in VCaP xenograft models. T-474 exhibited potent antitumor activity when orally administered at 5 mg/kg once daily [Treatment over control (T/C) = 23%, *P* < 0.01] for 21 days without severe body weight reduction (Figure 5A and 5B). Western blot analysis in parallel studies of mice treated for 3 days revealed significant reductions of STAT1 phosphorylation in VCaP tumors (Figure 5C). Consistent with *in vitro* observation, T-474 administration up-regulated c-Myc, MCM2 phosphorylation, Chk1 phosphorylation, and γ-H2AX, whereas it down-regulated p21 (Figure 5C). These results indicate that T-474 administration causes aberrant G1/S transition and activates the DDR machinery in VCaP xenografts, thereby exhibiting significant antitumor activity.

## DISCUSSION

In this study, we used two chemically distinct CDK8/19 inhibitors, both of which showed high selectivity against CDK8 and CDK19. These compounds provided us a means to precisely understand the biological phenomena dependent on CDK8/19 kinase activity. The data presented here show that inhibition of CDK8/19 by the compounds and dual depletion of CDK8/19 by siRNAs suppressed the proliferation of VCaP prostate cancer cells. The individual depletion of CDK8 or CDK19 did not have an obvious impact on cell proliferation. These results indicate that CDK8 and CDK19 may have complementary roles and that inhibition of both is required for suppressing the proliferation of VCaP cells.

Recently, several studies have explored the role of CDK8/19 in cancer by using small molecules that selectively inhibit CDK8/19 [19, 21, 36]. As CDK8 is amplified and functions as an oncogene in colorectal cancer through the regulation of Wnt/β-catenin signaling [17, 18, 37], the therapeutic potential of the small molecules has been actively tested in preclinical models of colorectal and other cancers [19, 36]. Additionally, cortistatin A, a natural product inhibitor of CDK8/19, has been shown to demonstrate antitumor activity in acute myelogenous leukemia (AML) cells [21]. Specifically, Pelish *et al.* reported that the increased activation of super-enhancer genes in AML cells by cortistatin A results in antileukemic activity [21]. In the current study, to characterize the mechanism by which CDK8/19 contributes to the proliferation of prostate cancer cells, we primarily utilized prostate cancer VCaP cells, which displayed the highest sensitivity to CDK8/19 inhibition among the prostate cancer cells tested. Given that VCaP cells show the highest expression of CDK19 among a large number of cell lines (approximately 1000 cell lines) in the Cancer Cell Line Encyclopedia (CCLE) database (https://portals.broadinstitute.org/ccle/home), it is plausible that overexpression of CDK19 plays a predominant role in prostate cancer cell proliferation. However, the CDK19 protein level in VCaP cells was high although comparable to that of LNCaP and 22Rv1 cells, which were insensitive to T-474 treatment. Factors other than CDK19 expression level are therefore likely involved in determining the sensitivity to CDK8/19 inhibitors.

We expect that the VCaP sensitivity is not due to an off-target effect of our compounds because the anti-proliferative effects were observed for two structurally distinct compounds, each of which has high kinase selectivity. Furthermore, the simultaneous depletion of CDK8 and CDK19 suppressed the proliferation of VCaP cells. However, we cannot completely deny the possibility of off-target effects in the absence of a rescue experiment using a CDK8/19 mutant refractory to T-474 or T-418 binding. Thus, our data suggest that inhibition of CDK8/19 by our compounds contributes to anti-proliferative activity in prostate cancer cells both *in vitro* and *in vivo*, although it remains possible that the effects may be influenced by the inhibition of a protein other than CDK8/19. Although only a limited number of prostate cancer cell lines are currently available, further investigation will be required to extend the generality of our findings.

Our results demonstrate that CDK8/19 prevents premature G1/S transition by modulating the gene expression of G1/S transition regulators in VCaP cells. Consistent with this finding, it has been reported that CDK8 positively regulates the p53-p21 pathway activation and thereby inhibits the G1/S transition [8]. However, this mechanism would not be expected to play an important role in VCaP cells, because these cells harbor an R248W mutation in the *TP53* gene, which is a gain-of-function mutation abrogating wild-type p53 activity [38]. In addition, a genetic screen in *Caenorhabditis elegans* also demonstrated that CDK8 module components are required for the control of entry into G0 during development [39]. Conversely, it has been suggested that CDK8 functions as a positive regulator of the G1/S transition through Wnt/β-catenin and/or E2F signaling [7, 13, 17]. These findings indicate that the CDK8/19 module contributes to regulation of the G1/S transition in a context-dependent manner.

Phosphoproteome and transcriptome analyses have revealed that CDK8/19 predominantly modulates the regulators of transcription [40]. Among the G1/S transition regulators tested in the current study, the expression of c-Myc was substantially affected by CDK8/19 inhibition. Notably, a previous report showed that CDK8 binds to the *MYC* promoter as a component of the Mediator complex, albeit with a positive impact on c-Myc transcription [17]. The increased c-Myc expression mediated by our CDK8/19 inhibitors may be linked to super-enhancer activation, as *MYC* is known to be a super-enhancer-associated gene [41]. However, one caveat for this interpretation is that *MYC* has not been confirmed to be SE-associated in VCaP cells; therefore, further study is needed to clarify this issue. Given that leukemic cells are sensitive to the dosage of super-enhancer-associated genes affected by CDK8/19 inhibition [21], the possibility exists that certain prostate cancer cells such as VCaP cells may also show vulnerability to the activation of super-enhancer-associated genes. In CDK8/19 inhibitor-sensitive prostate cancer cells, we found that c-Myc depletion prevents premature G1/S transition and the subsequent DDR resulting from CDK8/19 inhibition. Furthermore, we also showed that c-Myc upregulation and the defects in cell cycle control did not occur in CDK8/19 inhibitor-insensitive cells. The findings support the upregulation of the *MYC* gene as a critical molecular response to CDK8/19 inhibition in CDK8/19 inhibitor-sensitive cells. However, we cannot exclude the contribution of other factors to this process.

It has been reported that overexpression of c-Myc not only accelerates the G1/S transition but also induces DNA damage and a hypersensitivity to DNA-damaging agents [42–44]. The premature G1/S transition might enable cells to enter the S phase in the presence of unrepaired damage. In support of this mechanism, we found that CDK8/19 inhibitor-treated VCaP cells acquire DNA damage and are sensitive to multiple topoisomerase inhibitors. Furthermore, a previous report showed that cells expressing c-Myc at high levels undergo p53-dependent G2 arrest [44]. It has been also shown that the G2/M checkpoint against DNA damage is impaired in cells with p53^R248W^ [45]. Consistent with these reports, VCaP cells (which carry p53^R248W^) [38] did not undergo G2 arrest when c-Myc expression and DDR were induced by CDK8/19 inhibitors. The G2/M checkpoint impairment in c-Myc overexpressing cells is expected to cause uncontrolled proliferation without appropriate DNA repair. Notably, CDK8/19 inhibitors did not promote the proliferation of VCaP cells but rather led to cell death. Thus, the possibility exists that VCaP cells would be particularly vulnerable to the stress resulting from c-Myc-driven premature G1/S transition. Further investigation will allow us to better understand the mechanisms underlying their observed sensitivity to CDK8/19 inhibition.

DDR induces cell cycle arrest for the repair of errors in DNA or to trigger cell death, depending on the type of cell and the level of DNA damage. Although many studies have examined the cytoprotective role of ATR, a pro-apoptotic role of ATR has been also reported [46–48]. In our study, an ATR inhibitor clearly reversed the reduced cell viability of CDK8/19 inhibitor-treated cells. Further investigation will provide a better understanding of the means by which CDK8/19 inhibition triggers cell death via ATR and its downstream effectors.

In summary, our preclinical study demonstrates that CDK8/19 inhibition induces cell death in VCaP prostate cancer cells. Inhibition of CDK8/19 activity modulates the mRNA expression of G1/S transition regulators, leading to premature G1/S transition followed by DDR and cell death in an ATR-dependent manner. These results shed light on the importance of CDK8/19 kinase as a hub for integrating transcription regulation with cell cycle control in prostate cancer cells.

## MATERIALS AND METHODS

### Reagents

T-474 (1-methyl-8-((2-methylpyridin-3-yl)oxy)-4,5-dihydro-1H-thieno[3,4-g]indazole-6-carboxamide) and T-418 ((2E)-3-(4-(4-fluorophenyl)pyridin-3-yl)-N-(4-(2-(1,3,4-oxadiazol-2-yl)ethyl)phenyl)acrylamide) were synthesized by Takeda Pharmaceutical Company Limited (WO2012/008549, WO2001/074823) [49, 50]. KU-55933, NU7026, and VE-821 were obtained from Selleck Chemicals. IFNγ and Z-VAD were obtained from R&D Systems and Sigma-Aldrich, respectively.

### Cell lines and culture

Prostate cancer cell lines (22Rv1, DU 145, LnCaP, PC-3, and VCaP), multiple myeloma cell line (RPMI8226), and colorectal cancer cell line (SW480) were obtained from American Type Culture Collection (ATCC) in 2013. The cell lines were cultured at 37° C with 5% CO_2_ in the recommended medium supplemented with 10–20% FBS (Thermo Fisher Scientific). The cell lines were stocked after *Mycoplasma* testing (performed by the Central Institute for Experimental Animals) and used within 2 months after resuscitation. The ATCC uses short tandem repeat profiling for authentication of cell lines. No authentication was performed by the authors.

### Ligand displacement assay against CDK8 and CDK19 kinases

Ligand displacement assays were conducted with Tb-labeled anti-GST antibody (CisBio), 20 nmol/L Kinase Tracer 236 (Thermo Fisher), 20 nmol/L GST-CDK8/Cyclin C (CarnaBio) or GST-CDK19/Cyclin C (CarnaBio), and each compound. All components were diluted in the assay buffer containing 25 mmol/L HEPES, 10 mmol/L MgCl_2_, 2 mmol/L dithiothreitol, and 0.01% Tween-20. After 60 minutes of incubation at room temperature, time resolved-fluorescence resonance energy transfer was measured using an EnVision plate reader (PerkinElmer). IC_50_ values were calculated with the nonlinear least square method using XLfit software (IDBS).

### Recombinant kinome-wide selectivity profiling

Kinase selectivity of T-474 and T-418 was evaluated at 300 nmol/L using a panel of 456 kinases at DiscoveRx (KINOMEscan). The platform employs an active site-directed competition binding assay to quantitatively measure interactions between test compounds and kinases.

### Public gene expression data

The data for *CDK8* and *CDK19* mRNA expression were collected from public databases of the CCLE.

### Transfection and siRNA

Cells were transfected with siRNAs using Lipofectamine RNAi MAX (Invitrogen). CDK8 and CDK19 siRNAs were obtained from GE Dharmacon (OnTARGETplus). c-Myc siRNAs were obtained from Thermo Fisher Scientific (Silencer Select) or GE Dharmacon (OnTARGETplus). Cell Death Control siRNA was used as a positive control for siRNA transfection (Qiagen). The product information of the siRNAs is as follows: CDK8-1: J-003242-09; CDK8-2: J-003242-10; CDK8-3: J-003242-11; CDK8-4: J-003242-12; CDK19-1: J-004689-05; CDK19-2: J-004689-06; CDK19-3: J-004689-07; CDK19-4: J-004689-08; Myc-S1: s9129; Myc-S2: s9130; Myc-O1: J-003282-23; Myc-O2: J-003282-24; Myc-O3: J-003282-26.

### Reporter gene assay

The Wnt reporter gene assay was performed as previously described [51, 52]. The reporter gene activity was assessed using the Dual-Glo luciferase assay (Promega), according to the manufacturer’s protocol.

### Western blot

Western blot analysis was performed as previously described [53]. Antibodies against the following proteins were used for immunoblot: MCM2 (ab108935) and phospho-MCM2 (ab133243) (Abcam), p27 (610242; BD Transduction Laboratories), β-actin (4970), c-Myc (5605), phospho-Ser345-Chk1 (2348), cleaved PARP (5625), γ-H2AX (2577), GAPDH (2118), p21 (2947), STAT1 (9175), and phospho-Ser727-STAT1 (8826) (Cell Signaling Technology), phospho-Ser10-histone H3 (06–570; Millipore), Cdc6 (sc-9964), CDK8 (sc-1521), Cyclin B1 (sc-752), and FANCD2 (sc-20022) (Santa Cruz Biotechnology), and α-tubulin (T9026) and CDK19 (HPA007053) (Sigma-Aldrich). α-tubulin, β-actin, and GAPDH were used as loading controls.

### Flow cytometric analysis

Cells were harvested and fixed with 70% ice-cold ethanol for 30 minutes at 4° C. After washing with PBS, the cells were incubated with PBS containing 1 mg/mL RNase for 30 minutes at 37° C. After washing with PBS again, the cells were stained with 50 μg/mL propidium iodide for 30 minutes at 4° C. EdU pulse-chase analysis was performed using the CLICK-iT Plus EdU flow cytometry assay kit (Thermo Fisher Scientific). Analyses were performed on a FACS Calibur (BD Biosciences).

### Quantitative PCR

RNA was extracted using the miRNeasy kit (Qiagen). cDNA was synthesized using the Verso cDNA kit (Thermo Scientific). Real-time quantitative PCR (qPCR) was performed on an ABI StepOne real-time PCR system (Applied Biosystems) using TaqMan gene expression assays for *AR* (Hs00171172_m1), *CDK19* (Hs01039930_g1), and *KLK3* (Hs02576345_m1) (Applied Biosystems). Relative gene expression was calculated using the ΔΔC_T_ method following the manufacturer’s instructions. The expression ratios of the indicated genes were normalized by the *GAPDH* expression in each cell line.

### Microarray analysis

Total RNA was extracted using the RNeasy Mini kit (Qiagen). Preparation of the cDNA and cRNA, hybridization, and microarray scanning were performed according to the manufacturer’s protocols (Affymetrix Inc.). The biotinylated cRNA was hybridized to Affymetrix U133 Plus 2 human genome arrays. The captured signals were normalized to the median expression level using the GeneSpring software package (Agilent Technology) and the normalized data were filtered by present/absent calls and the expression level. Pathway analysis was performed with PAGE [54] using the NCBI BioSystems Database [55].

### Cell viability assay

Cell viability was assessed using the CellTiter-Glo luminescent cell viability assay (Promega) or CyQuant Direct (Thermo Fisher Scientific), according to the manufacturer’s protocol. Sigmoidal dose-response (variable slope) curves were fitted using non-linear regression analysis (GraphPad Prism version 6; GraphPad Software).

### Caspase 3/7 assay

Caspase 3/7 activity was assessed using the Caspase-Glo 3/7 assay (Promega), according to the manufacturer’s protocol.

### Animal study

Suspensions of VCaP prostate cancer cells (1 × 10^6^ cells/site) were subcutaneously implanted into the right flank of 6-week-old male SCID mice (C.B17/Icr-scid/scid Jcl, CLEA Japan). Tumor volumes were calculated as volume = L × l^2^ × 1/2, where L was taken to be the longest diameter across the tumor and l was taken to be the corresponding perpendicular distance. Body weight was also measured. For the antitumor activity test, when the tumor mass was approximately 200 mm^3^, mice were sorted into treatment groups (*N* = 6/group) such that the mean tumor volume and body weight between groups were similar. Tumors were monitored and mice were euthanized when an endpoint was reached, defined as tumor volume larger than 2000 mm^3^, severe ataxia, body weight loss (>20% compared to the body weight on the day of randomization), or study end, whichever came first. From the day of randomization, T-474 dissolved in distilled water containing 0.5% methyl cellulose was orally administered to mice bearing a xenograft for 21 days. Treatment over control (T/C, %), an index of antitumor activity, was calculated by comparison of the mean change in tumor volume over the treatment period for the control and treated groups. For western blot analysis, tumors were homogenized using a Lysing Matrix I tube (MP Biomedicals) in Lysing solution [10% glycerol, 1% sodium dodecyl sulfate, 62.5 mmol/L Tris-HCl (pH 7.5), protease inhibitors, and phosphatase inhibitors (cOmplete mini and PhosSTOP, Sigma-Aldrich)]. The mice were housed and maintained in accordance with institutional guidelines established by the Institutional Animal Care and Use Committee, in a facility accredited by the American Association for Accreditation of Laboratory Animal Care. The animal experimental protocols were approved by the Institutional Animal Care and Use Committee.

### Statistical analysis

Groups were compared by Bonferroni’s multiple comparison test using GraphPad Prism software (version 6). To assess *in vivo* antitumor activity, Welch’s *t* test was performed with treatment over control (T/C, %) values using Excel (Microsoft). Differences were considered significant at *P* < 0.05.

## SUPPLEMENTARY MATERIALS FIGURES AND TABLES







## Abbreviations

Pol II: RNA polymerase II
PAGE: parametric analysis of gene set enrichment
AR: androgen receptor
DDR: DNA damage response
CCLE: Cancer Cell Line Encyclopedia
ATCC: American Type Culture Collection
qPCR: real-time quantitative PCR
ETP: etoposide
DOX: doxorubicin
CHX: cycloheximide

## References

[1] Malumbres M, Barbacid M (2009). Cell cycle, CDKs and cancer: a changing paradigm. Nat Rev Cancer.

[2] Loyer P, Trembley JH, Katona R, Kidd VJ, Lahti JM (2005). Role of CDK/cyclin complexes in transcription and RNA splicing. Cell Signal.

[3] Galbraith MD, Donner AJ, Espinosa JM (2010). CDK8: a positive regulator of transcription. Transcription.

[4] Allen BL, Taatjes DJ (2015). The Mediator complex: a central integrator of transcription. Nat Rev Mol Cell Biol.

[5] Whyte WA, Orlando DA, Hnisz D, Abraham BJ, Lin CY, Kagey MH, Rahl PB, Lee TI, Young RA (2013). Master transcription factors and mediator establish super-enhancers at key cell identity genes. Cell.

[6] Larochelle S, Merrick KA, Terret ME, Wohlbold L, Barboza NM, Zhang C, Shokat KM, Jallepalli PV, Fisher RP (2007). Requirements for Cdk7 in the assembly of Cdk1/cyclin B and activation of Cdk2 revealed by chemical genetics in human cells. Mol Cell.

[7] Szilagyi Z, Gustafsson CM (2013). Emerging roles of Cdk8 in cell cycle control. Biochim Biophys Acta.

[8] Donner AJ, Szostek S, Hoover JM, Espinosa JM (2007). CDK8 is a stimulus-specific positive coregulator of p53 target genes. Mol Cell.

[9] Morris EJ, Ji JY, Yang F, Di Stefano L, Herr A, Moon NS, Kwon EJ, Haigis KM, Naar AM, Dyson NJ (2008). E2F1 represses beta-catenin transcription and is antagonized by both pRB and CDK8. Nature.

[10] Niehrs C, Acebron SP (2012). Mitotic and mitogenic Wnt signalling. EMBO J.

[11] Wong JV, Dong P, Nevins JR, Mathey-Prevot B, You L (2011). Network calisthenics: control of E2F dynamics in cell cycle entry. Cell Cycle.

[12] Xu D, Li CF, Zhang X, Gong Z, Chan CH, Lee SW, Jin G, Rezaeian AH, Han F, Wang J, Yang WL, Feng ZZ, Chen W (2015). Skp2-macroH2A1-CDK8 axis orchestrates G2/M transition and tumorigenesis. Nat Commun.

[13] Zhao J, Ramos R, Demma M (2013). CDK8 regulates E2F1 transcriptional activity through S375 phosphorylation. Oncogene.

[14] Bragelmann J, Klumper N, Offermann A, von Massenhausen A, Bohm D, Deng M, Queisser A, Sanders C, Syring I, Merseburger AS, Vogel W, Sievers E, Vlasic I (2017). Pan-cancer analysis of the Mediator complex transcriptome identifies CDK19 and CDK8 as therapeutic targets in advanced prostate cancer. Clin Cancer Res.

[15] Syring I, Klumper N, Offermann A, Braun M, Deng M, Boehm D, Queisser A, von Massenhausen A, Bragelmann J, Vogel W, Schmidt D, Majores M, Schindler A (2016). Comprehensive analysis of the transcriptional profile of the Mediator complex across human cancer types. Oncotarget.

[16] Broude EV, Gyorffy B, Chumanevich AA, Chen M, McDermott MS, Shtutman M, Catroppo JF, Roninson IB (2015). Expression of CDK8 and CDK8-interacting Genes as Potential Biomarkers in Breast Cancer. Curr Cancer Drug Targets.

[17] Firestein R, Bass AJ, Kim SY, Dunn IF, Silver SJ, Guney I, Freed E, Ligon AH, Vena N, Ogino S, Chheda MG, Tamayo P, Finn S (2008). CDK8 is a colorectal cancer oncogene that regulates beta-catenin activity. Nature.

[18] Seo JO, Han SI, Lim SC (2010). Role of CDK8 and beta-catenin in colorectal adenocarcinoma. Oncol Rep.

[19] Dale T, Clarke PA, Esdar C, Waalboer D, Adeniji-Popoola O, Ortiz-Ruiz MJ, Mallinger A, Samant RS, Czodrowski P, Musil D, Schwarz D, Schneider K, Stubbs M (2015). A selective chemical probe for exploring the role of CDK8 and CDK19 in human disease. Nat Chem Biol.

[20] Porter DC, Farmaki E, Altilia S, Schools GP, West DK, Chen M, Chang BD, Puzyrev AT, Lim CU, Rokow-Kittell R, Friedhoff LT, Papavassiliou AG, Kalurupalle S (2012). Cyclin-dependent kinase 8 mediates chemotherapy-induced tumor-promoting paracrine activities. Proc Natl Acad Sci U S A.

[21] Pelish HE, Liau BB, Nitulescu II, Tangpeerachaikul A, Poss ZC, Da Silva DH, Caruso BT, Arefolov A, Fadeyi O, Christie AL, Du K, Banka D, Schneider EV (2015). Mediator kinase inhibition further activates super-enhancer-associated genes in AML. Nature.

[22] Saeed K, Ostling P, Bjorkman M, Mirtti T, Alanen K, Vesterinen T, Sankila A, Lundin J, Lundin M, Rannikko A, Nordling S, Mpindi JP, Kohonen P (2015). Androgen receptor-interacting protein HSPBAP1 facilitates growth of prostate cancer cells in androgen-deficient conditions. Int J Cancer.

[23] Bancerek J, Poss ZC, Steinparzer I, Sedlyarov V, Pfaffenwimmer T, Mikulic I, Dolken L, Strobl B, Muller M, Taatjes DJ, Kovarik P (2013). CDK8 kinase phosphorylates transcription factor STAT1 to selectively regulate the interferon response. Immunity.

[24] Galbraith MD, Allen MA, Bensard CL, Wang X, Schwinn MK, Qin B, Long HW, Daniels DL, Hahn WC, Dowell RD, Espinosa JM (2013). HIF1A employs CDK8-mediator to stimulate RNAPII elongation in response to hypoxia. Cell.

[25] Sclafani RA, Holzen TM (2007). Cell cycle regulation of DNA replication. Annu Rev Genet.

[26] Besson A, Dowdy SF, Roberts JM (2008). CDK inhibitors: cell cycle regulators and beyond. Dev Cell.

[27] Shiloh Y (2003). ATM and related protein kinases: safeguarding genome integrity. Nat Rev Cancer.

[28] Charrier JD, Durrant SJ, Golec JM, Kay DP, Knegtel RM, MacCormick S, Mortimore M, O’Donnell ME, Pinder JL, Reaper PM, Rutherford AP, Wang PS, Young SC (2011). Discovery of potent and selective inhibitors of ataxia telangiectasia mutated and Rad3 related (ATR) protein kinase as potential anticancer agents. J Med Chem.

[29] Reaper PM, Griffiths MR, Long JM, Charrier JD, Maccormick S, Charlton PA, Golec JM, Pollard JR (2011). Selective killing of ATM- or p53-deficient cancer cells through inhibition of ATR. Nat Chem Biol.

[30] Ohashi A, Ohori M, Iwai K, Nakayama Y, Nambu T, Morishita D, Kawamoto T, Miyamoto M, Hirayama T, Okaniwa M, Banno H, Ishikawa T, Kandori H (2015). Aneuploidy generates proteotoxic stress and DNA damage concurrently with p53-mediated post-mitotic apoptosis in SAC-impaired cells. Nat Commun.

[31] Nitiss JL (2002). DNA topoisomerases in cancer chemotherapy: using enzymes to generate selective DNA damage. Curr Opin Investig Drugs.

[32] Smart DJ, Halicka HD, Schmuck G, Traganos F, Darzynkiewicz Z, Williams GM (2008). Assessment of DNA double-strand breaks and gammaH2AX induced by the topoisomerase II poisons etoposide and mitoxantrone. Mutat Res.

[33] Sears RC (2004). The life cycle of C-myc: from synthesis to degradation. Cell Cycle.

[34] Zeller KI, Jegga AG, Aronow BJ, O’Donnell KA, Dang CV (2003). An integrated database of genes responsive to the Myc oncogenic transcription factor: identification of direct genomic targets. Genome Biol.

[35] Ahuja AK, Jodkowska K, Teloni F, Bizard AH, Zellweger R, Herrador R, Ortega S, Hickson ID, Altmeyer M, Mendez J, Lopes M (2016). A short G1 phase imposes constitutive replication stress and fork remodelling in mouse embryonic stem cells. Nat Commun.

[36] Poss ZC, Ebmeier CC, Odell AT, Tangpeerachaikul A, Lee T, Pelish HE, Shair MD, Dowell RD, Old WM, Taatjes DJ (2016). Identification of Mediator Kinase Substrates in Human Cells using Cortistatin A and Quantitative Phosphoproteomics. Cell Rep.

[37] Clarke PA, Ortiz-Ruiz MJ, TePoele R, Adeniji-Popoola O, Box G, Court W, Czasch S, El Bawab S, Esdar C, Ewan K, Gowan S, De Haven Brandon A, Hewitt P (2016). Assessing the mechanism and therapeutic potential of modulators of the human Mediator complex-associated protein kinases. Elife.

[38] Adler AS, McCleland ML, Truong T, Lau S, Modrusan Z, Soukup TM, Roose-Girma M, Blackwood EM, Firestein R (2012). CDK8 maintains tumor dedifferentiation and embryonic stem cell pluripotency. Cancer Res.

[39] Sigal A, Rotter V (2000). Oncogenic mutations of the p53 tumor suppressor: the demons of the guardian of the genome. Cancer Res.

[40] Clayton JE, van den Heuvel SJ, Saito RM (2008). Transcriptional control of cell-cycle quiescence during C. elegans development. Dev Biol.

[41] Loven J, Hoke HA, Lin CY, Lau A, Orlando DA, Vakoc CR, Bradner JE, Lee TI, Young RA (2013). Selective inhibition of tumor oncogenes by disruption of super-enhancers. Cell.

[42] Felsher DW, Bishop JM (1999). Transient excess of MYC activity can elicit genomic instability and tumorigenesis. Proc Natl Acad Sci U S A.

[43] Herold S, Herkert B, Eilers M (2009). Facilitating replication under stress: an oncogenic function of MYC?. Nat Rev Cancer.

[44] Karn J, Watson JV, Lowe AD, Green SM, Vedeckis W (1989). Regulation of cell cycle duration by c-myc levels. Oncogene.

[45] Song H, Hollstein M, Xu Y (2007). p53 gain-of-function cancer mutants induce genetic instability by inactivating ATM. Nat Cell Biol.

[46] Marechal A, Zou L (2013). DNA damage sensing by the ATM and ATR kinases. Cold Spring Harb Perspect Biol.

[47] Pabla N, Huang S, Mi QS, Daniel R, Dong Z (2008). ATR-Chk2 signaling in p53 activation and DNA damage response during cisplatin-induced apoptosis. J Biol Chem.

[48] Norbury CJ, Zhivotovsky B (2004). DNA damage-induced apoptosis. Oncogene.

[49] Fujimoto J, Hirayama T, Hirata Y, Hikichi Y, Murai S, Hasegawa M, Hasegawa Y, Yonemori K, Hata A, Aoyama K, Cary DR (2017). Studies of CDK8/19 inhibitors: Discovery of novel and selective CDK8/19 dual inhibitors and elimination of their CYP3A4 time-dependent inhibition potential. Bioorg Med Chem.

[50] Ono K, Banno H, Okaniwa M, Hirayama T, Iwamura N, Hikichi Y, Murai S, Hasegawa M, Hasegawa Y, Yonemori K, Hata A, Aoyama K, Cary DR (2017). Design and synthesis of selective CDK8/19 dual inhibitors: Discovery of 4,5-dihydrothieno[3’,4’:3,4]benzo[1,2-d]isothiazole derivatives. Bioorg Med Chem.

[51] Fukuda Y, Sano O, Kazetani K, Yamamoto K, Iwata H, Matsui J (2016). Tubulin is a molecular target of the Wnt-activating chemical probe. BMC Biochem.

[52] Molenaar M, van de Wetering M, Oosterwegel M, Peterson-Maduro J, Godsave S, Korinek V, Roose J, Destree O, Clevers H (1996). XTcf-3 transcription factor mediates beta-catenin-induced axis formation in Xenopus embryos. Cell.

[53] Nakamura A, Naito M, Tsuruo T, Fujita N (2008). Freud-1/Aki1, a novel PDK1-interacting protein, functions as a scaffold to activate the PDK1/Akt pathway in epidermal growth factor signaling. Mol Cell Biol.

[54] Kim SY, Volsky DJ (2005). PAGE: parametric analysis of gene set enrichment. BMC Bioinformatics.

[55] Geer LY, Marchler-Bauer A, Geer RC, Han L, He J, He S, Liu C, Shi W, Bryant SH (2010). The NCBI BioSystems database. Nucleic Acids Res.

