# Supplementary material for: CDK8/19 inhibition induces premature G1/S transition and ATR-dependent cell death in prostate cancer cells
Source: Oncotarget. 2018 Feb 6;9(17):13474–87. doi: 10.18632/oncotarget.24414 (PMC5862592; doi:10.18632/oncotarget.24414)
Supplement: Supplementary file 1 [file oncotarget-09-13474-s001.pdf]

CDK8/19 inhibition induces premature G1/S transition and ATR-dependent cell death in prostate cancer cells

SUPPLEMENTARY MATERIALS

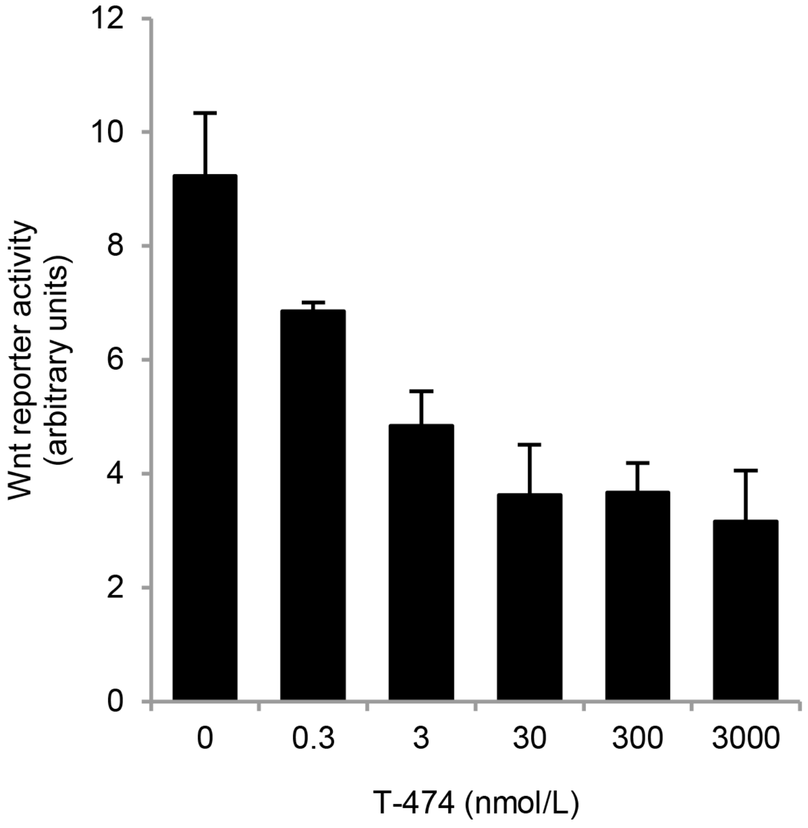

Supplementary Figure 1: Suppression of β-catenin activity by CDK8/19 inhibitors. SW480 cells were transfected with a *Wnt* reporter gene and internal control plasmids. After transfection for 24 hours, cells were treated with T-474 at the indicated concentrations for 24 hours. Luciferase activity was measured ($N = 3$, mean with *SD*).

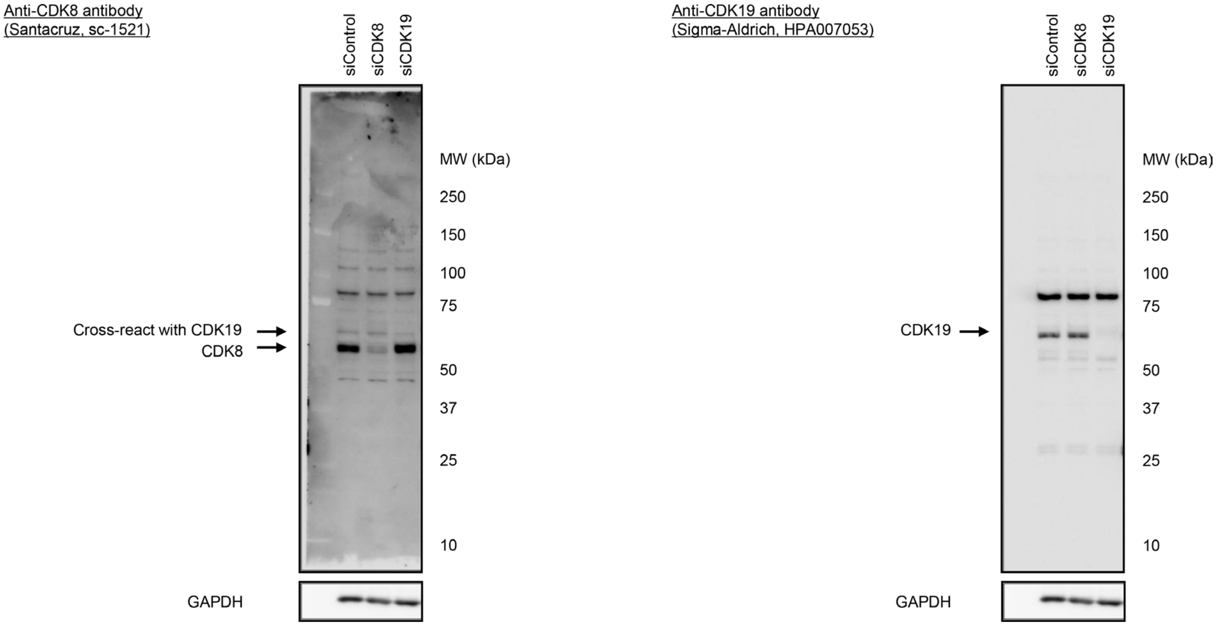

Supplementary Figure 2: Validation of anti-CDK8 and anti-CDK19 antibodies for western blot. VCaP cells were transfected with siRNA as indicated for 72 hours. Cell lysates were analyzed by western blot. Arrows indicate the expected position of bands derived from CDK8 or CDK19, respectively.

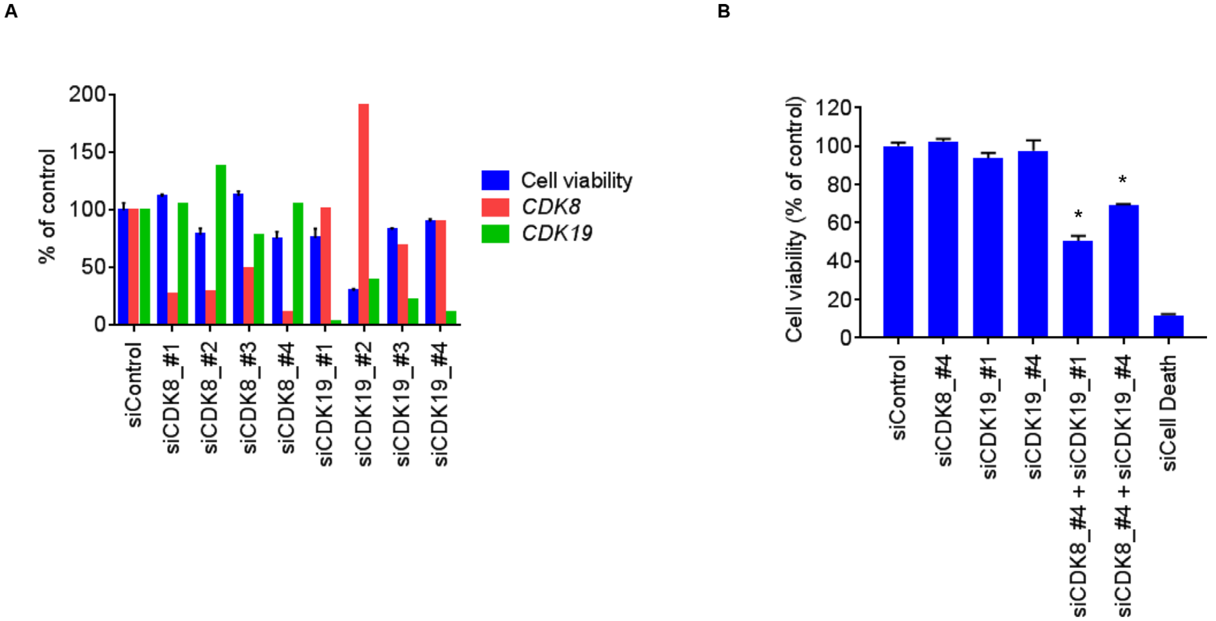

Supplementary Figure 3: Anti-proliferative activity of CDK8/19 knockdown. (**A**) VCaP cells were successively transfected with siRNA as indicated every 3-4 days for 14 days. Blue bars, cell viability ($N = 3$, mean with *SD*). Red and green bars, qPCR analysis of cell lysates ($N = 2$, mean). (**B**) VCaP cells were transfected with siRNA as indicated for 7 days and then cell viability was measured ($N = 3$, mean with *SD*). $^*P < 0.0001$ (compared with control siRNA-treated cells).

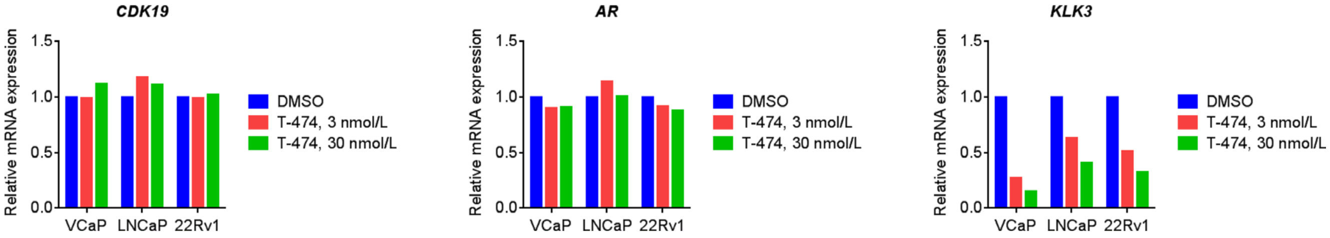

Supplementary Figure 4: Effects of CDK8/19 inhibition on AR signaling. VCaP, LNCaP, or 22Rv1 cells were treated with T-474 as indicated for 24 hours. Cell lysates were analyzed by qPCR ($N = 2$, mean).

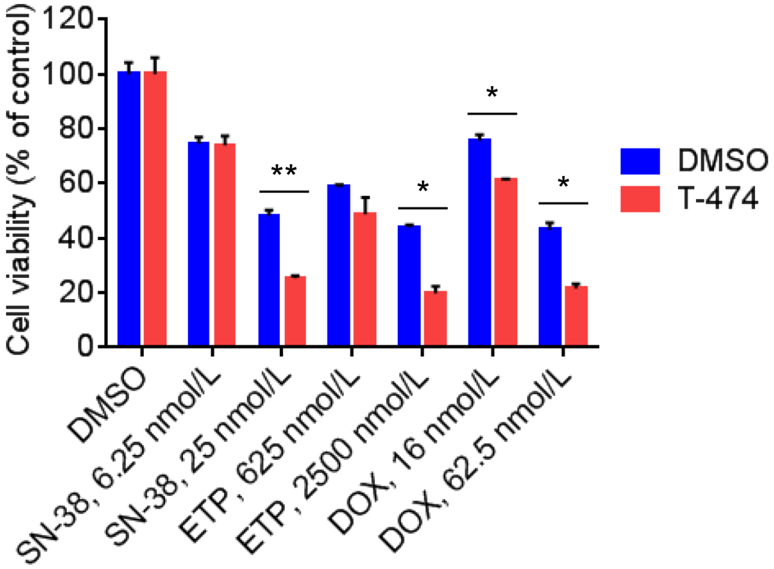

Supplementary Figure 5: Anti-proliferative activity of combination treatment with CDK8/19 inhibitors and topoisomerase inhibitors. VCaP cells were treated with 30 nmol/L T-474 together with SN-38, etoposide (ETP), or doxorubicin (DOX) as indicated for 4 days. Cell viability was measured and normalized against DMSO or T-474-treated cells (mean with *SD*; $N = 3$). $^{*}P < 0.001$; $^{**}P < 0.0001$.

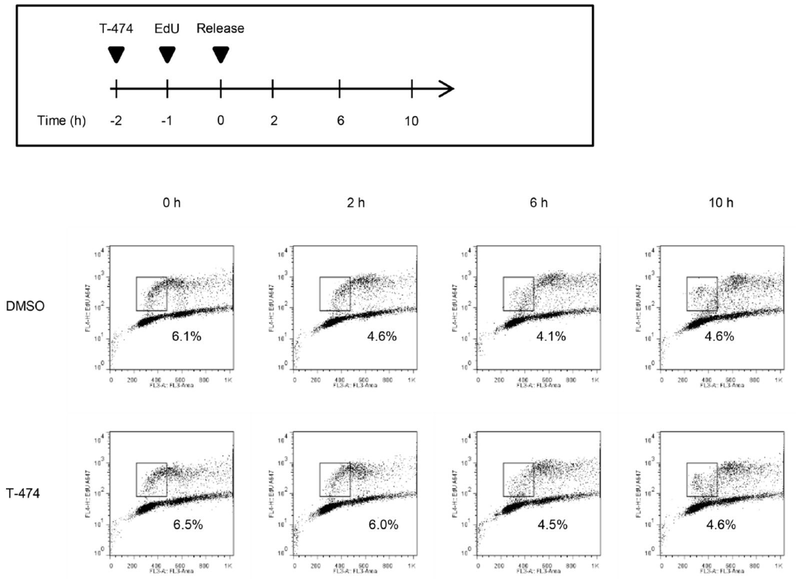

Supplementary Figure 6: Effects of CDK8/19 inhibition on S phase progression. VCaP cells were treated with 30 nmol/L T-474 for 1 hour and then with 10 µmol/L EdU for 1 hour. The cells were washed in EdU-free medium containing 30 nmol/L T-474. At 0, 2, 6, or 10 hours after release of EdU, the cell cycle profile was analyzed using a flow cytometer.

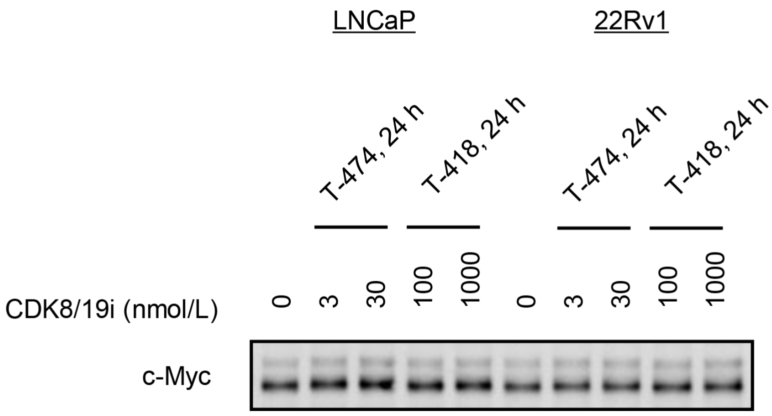

Supplementary Figure 7: Effects of CDK8/19 inhibition on c-Myc expression in CDK8/19-insensitive cells. LNCaP or 22Rv1 cells were treated with T-474 or T-418 as indicated for 24 hours. Cell lysates were analyzed by western blot (the same lysates as in Figure 2D).

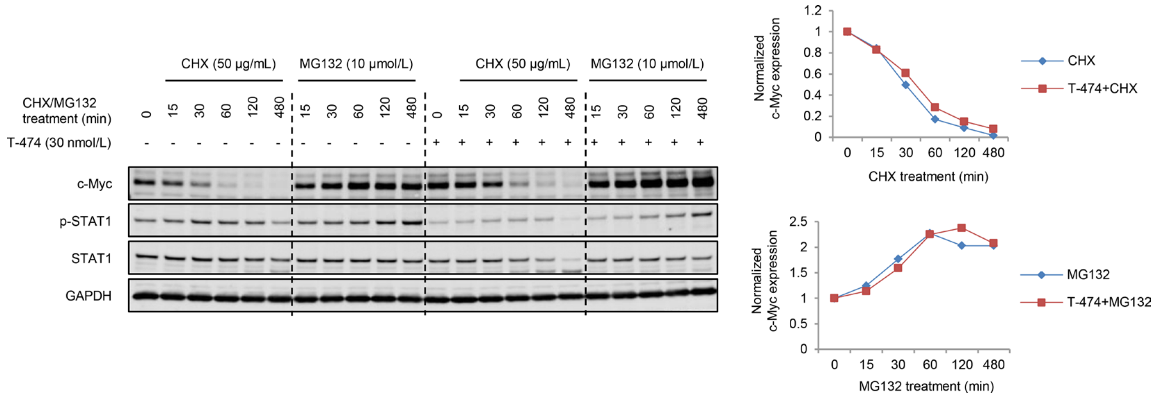

Supplementary Figure 8: Effects of CDK8/19 inhibition on turnover of c-Myc protein. VCaP cells were pre-treated with 30 nmol/L T-474 for 3 days and then treated with 50 µg/mL CHX or 10 µmol/L MG132 as indicated. Cell lysates were analyzed by western blot. Line graphs represent densitometric analysis of c-Myc divided by GAPDH, normalized to DMSO or T-474-treated controls.

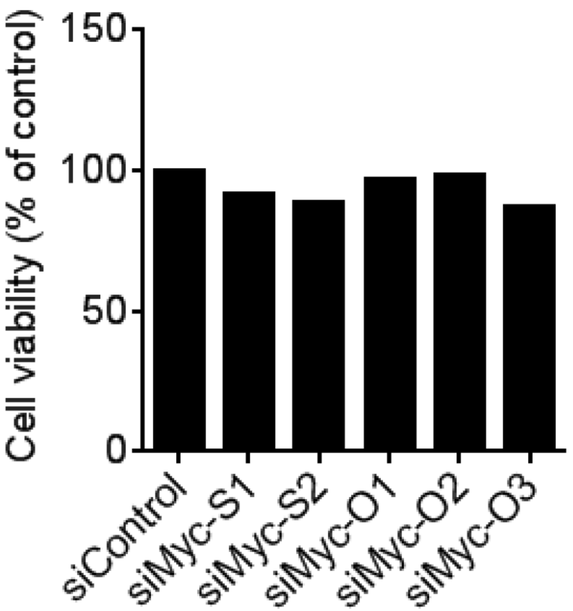

Supplementary Figure 9: Effects of c-Myc depletion on cell proliferation. VCaP cells were transfected with siRNA as indicated for 96 hours and then cell viability was measured (*N* = 2, mean).

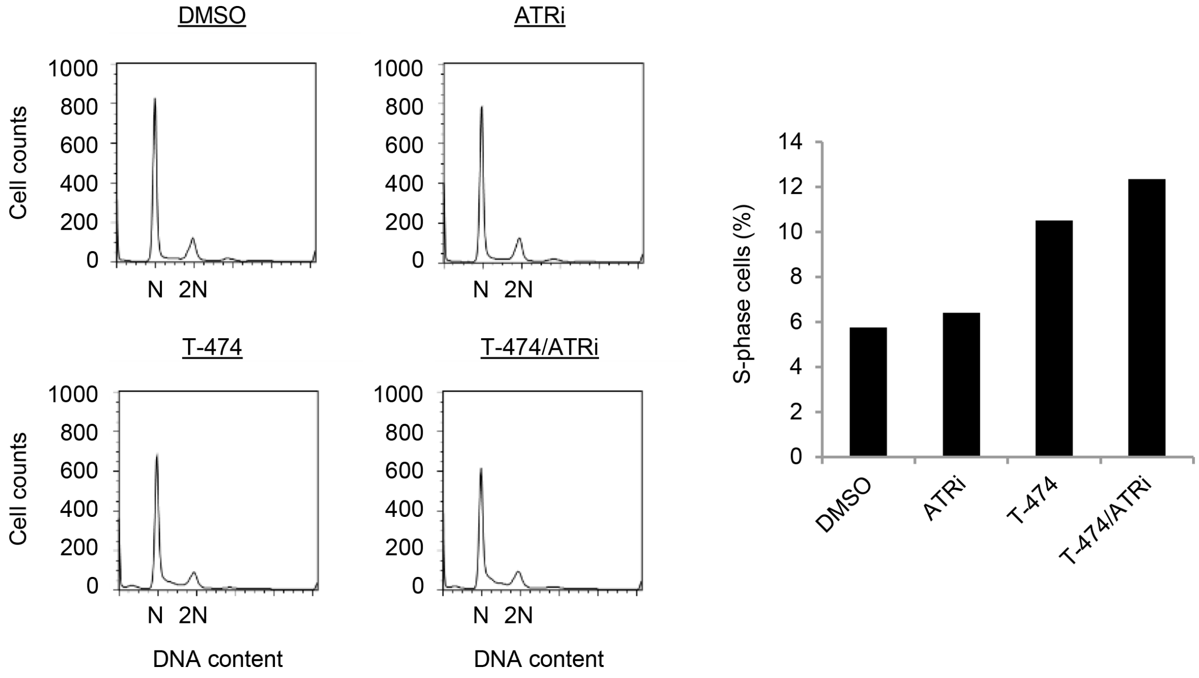

Supplementary Figure 10: Effects of CDK8/19 inhibition or ATR inhibition on the cell cycle. VCaP cells were treated with 30 nmol/L T-474 and/or 1000 nmol/L ATRi (VE-821) for 24 hours. The cell cycle profile was analyzed using a flow cytometer. Bars show the population of cells in S phase (*N* = 2, mean).

Supplementary Table 1: Kinase profiling of T-474. See Supplementary_Table_1

Supplementary Table 2: Kinase profiling of T-418. See Supplementary_Table_2
